# Tracking changes in autonomic function by coupled analysis of wavelet-based dispersion of heart rate variability and gastrointestinal symptom severity in individuals with hypermobile Ehlers–Danlos syndrome

**DOI:** 10.3389/fneur.2024.1499582

**Published:** 2025-01-15

**Authors:** Sarah A. Mathena, Robert M. Allen, Christina Laukaitis, Jennifer G. Andrews

**Affiliations:** ^1^ARID Laboratory, Department of Pediatrics, College of Medicine, University of Arizona, Tucson, AZ, United States; ^2^Raytheon Technologies corporation (RTX), Tucson, AZ, United States; ^3^Stephens Family Clinical Research Institute, Carle Health, Urbana, IL, United States; ^4^Carle Illinois College of Medicine, University of Illinois, Urbana, IL, United States; ^5^Carl R. Woese Institute for Genomic Biology, University of Illinois, Urbana, IL, United States

**Keywords:** Ehlers–Danlos syndrome, autonomic dysfunction, heart rate variability, wavelet, gastrointestinal, biometrics

## Abstract

**Introduction:**

People with hypermobile Ehlers–Danlos syndrome (hEDS) experience multisystemic dysfunction with varying severity and unpredictability of flare occurrence. Cohort studies suggest that individuals with hEDS have a higher risk for autonomic dysfunction. The gold standard for assessing autonomic function, clinically, is the heart rate variability (HRV) assessment from 24-h Holter monitor electrocardiogram data, but this is expensive and can only be performed in short durations. Since their advent, biometric devices have been a non-invasive method for tracking HRV to assess autonomic function. This study aimed to understand the intra- and inter-individual variability in autonomic function and to associate this variability with gastrointestinal symptoms in individuals with hEDS using wearable devices.

**Methods:**

We studied 122 days of biometric device data from 26 individuals, including 35 days highlighted as high gastrointestinal (GI) dysfunction and 48 days as low GI dysfunction. Utilizing wavelet analysis to assess the frequency domains of heart rate signals, we compared participants’ HRV data for high, low, very low (VLF), and ultralow (ULF) frequency domains associated with physiological differences.

**Results:**

We found a significant difference between the VLF and ULF signals on high-GI symptom days compared with low-symptoms days for 92 and 76% of the signals sampled, respectively.

**Discussion:**

Our pilot data show a change in HRV for individuals with hEDS experiencing a flare day for a single-body system. Future research will focus on evaluating the relationship between longitudinal multisystemic symptom severity fluctuations and HRV.

## 1 Introduction

Ehlers–Danlos syndrome (EDS) is a group of 13 inherited connective tissue disorders, the most common type of which is hypermobile Ehlers–Danlos syndrome (hEDS), which does not have an established genetic etiology (1). Patients with hEDS are diagnosed using clinical criteria comprised of an assessment of generalized hypermobility in nine joints plus two of the following: (a) a minimum of 5 of 12 objective signs; (b) a first-degree family member diagnosed using the 2017 criteria; and/or (c) chronic pain or recurrent joint instability for at least 3 months, and all other differential diagnoses, including other types of EDS ruled out (1, 2). Individuals who do not meet the generalized hypermobility or minimum objective signs criteria but have other differential diagnoses ruled out, are considered to be on the same disease spectrum and are diagnosed with hypermobile spectrum disorder (HSD) (3). hEDS accounts for approximately 90% of all EDS diagnoses, with prevalence estimates at 1 in 500 people (4); however, prevalence for hEDS and HSD is posited in as many as 1 in 100 people (3, 5).

People with hEDS experience dysfunction across multiple body systems resulting in a wide range of intermittent symptoms of varying severity, both within and across affected individuals. Signs and symptoms in people with hEDS include chronic pain, frequent joint subluxations and dislocations, sleep disturbance, fatigue, immune and inflammatory issues, gastrointestinal (GI) issues, and orthostatic dysfunction (1, 6). Two of the most prominent and interrelated patient complaints involve GI and orthostatic intolerance (OI) symptoms. This is supported by reports showing that a hEDS diagnosis increases the risk for autonomic dysfunction (AD) (7), including GI dysfunction (8, 9) and orthostatic issues (10). GI dysfunction in hEDS patients is frequently comorbidly reported with OI, and the absence of organic etiology indicates that the GI symptoms in hEDS patients are also attributable to AD (11).

While there are many manifestations of AD, orthostatic disturbances are highly studied and discussed. These are very common in people with hEDS, with 80–94% of people reporting orthostatic disturbances either through self-reported clinical symptom scales or as measured objective signs (12–14). Orthostatic problems are variable and are classified as orthostatic intolerance when signs and symptoms are present but do not meet the full criteria for postural orthostatic tachycardia syndrome (POTS). Still, for simplicity, we will refer to any degree of orthostatic intolerance as OI. OI is directly attributed to failures in the autonomic nervous system (ANS) in maintaining homeostasis. These ANS failures can present in multiple body systems and are functional, as indicated by fluctuating periods of severity within and between patient populations, which makes the diagnosis and management of AD clinically complicated (15, 16).

The exact prevalence of GI symptoms in people with hEDS is unknown due to the challenges inherent in diagnosing functional disorders (17), but one report shows that up to 62% of hEDS patients often receive an IBS diagnosis prior to their hEDS diagnosis (18). Furthermore, in another cohort study, 63% of patients had at least one GI symptom at the time of diagnosis (19) and one-third of all hEDS patients are referred to a GI clinic for management of symptoms (20). OI and GI symptoms are frequently comorbid in hEDS patients, and the absence of organic etiology for GI symptoms in hEDS patients presents the potential that those symptoms are attributable to AD (21, 22). hEDS patients with POTS have an increase in GI diagnoses and symptoms compared to POTS-negative hEDS patients, including increases in IBS (59% vs. 51%) and functional gastroduodenal disorders (75% vs. 67%) (18). GI and OI symptoms have a high impact on physical quality of life and pain, leading to reduced health outcomes and increased psychological stress (12, 21, 23).

People diagnosed with hEDS undergo a protracted diagnostic odyssey consisting of evaluation by an average of 15.6 different provider types, with nearly all (99.8%) reporting initially receiving at least one alternate diagnosis with an average of 10.45 codiagnoses for hEDS-associated signs and symptoms. Unfortunately, the time to diagnosis remains high at 10.4 years, with the majority receiving the diagnosis from subspecialists (77%), leaving many individuals undiagnosed and without a pathway for diagnosis (24).

Individuals who are embroiled within this pathway or who are postdiagnosis but still learning how best to manage their symptoms struggle with managing bouts of symptom exacerbation. Like other chronic disease symptoms, GI dysfunction often presents as intermittent, recurring events (i.e., flare-ups) that are not directly tied to known causes such as dietary or hormonal changes (25), making management of symptoms challenging for the patient (26). Reports of alternating bowel symptoms in hEDS patients further complicate this.

There has been a recent surge in wearable biometric devices marketed to the public. These provide an affordable means for self-monitoring previously restricted to the clinical realm. The dominant device type allows measurement of autonomic responses, including heart rate variability (HRV), breathing rate, pulse, oxygen saturation, movement, and many more emerging metrics. Recent data demonstrate physician use of these types of data to facilitate monitoring and management of disease efficacy, which becomes critical in multisystemic chronic conditions with a lack of providers, such as hEDS (27). Furthermore, the etiology of many of the reported complaints in hEDS patients is unknown, and autonomic failures to maintain homeostasis appropriately are accepted theories (28).

Autonomic function is evaluated using analysis of heart rate variability (HRV). Adaptations seen as fluctuations in heart rate (HR) are, essentially, differences in the time between beats [respiratory rate (RR) interval]. Having highly variable responses is consistent with having a normal and functional ANS. HRV is highly individualized, even among healthy populations, so analysis of functionality and fluctuations in HRV is best tested in individuals and populations over time (29); more specifically, fluctuations in HRV around a mean over time can be mathematically assessed to estimate the overall functionality of the ANS in an individual. The ANS consists of a system of excitatory and inhibitory signals that can interact synergistically or antagonistically. Thus, the RR signal can be analyzed as a composite of various co-occurring signals that can be mathematically separated or decomposed into distinct frequency domains representing the various ANS functions defined by the number of full signal cycles seen within a discrete unit of time or hertz (Hz).

Although identifying the contributions to symptoms flare-ups is difficult for the patients, identifying *when* the flare-up is occurring is easily discernible by patients and generally occurs for at least 24 h. Therefore, we proposed to pilot the tracking of autonomic function and occurrence of symptoms in hEDS patients over time using commercially available wearable biometric devices and daily self-report of symptom occurrence and severity to determine whether there is a predictable correlation that could be used for self-management of future flares. Given the strong co-occurrence of OI and GI symptoms in hEDS patients, we, limited this study to assessing the relationship between GI symptoms and HRV patterns. This study aims to establish methods for generating HRV metrics in a sample of individuals with hEDS and determining associations with GI symptom fluctuations.

The gold standard for assessing autonomic function, clinically, is the HRV, measuring the changes in time intervals between consecutive heartbeats from 24-h Holter monitor electrocardiogram (ECG) data (30). However, this is expensive and can only be performed in short durations. Technological improvements in biometric devices have increased sampling rates and increased their viability as a proxy for more expensive ECG devices in health studies, including for HRV (31, 32). Lower HRV is frequently associated with individuals with chronic conditions (33, 34) and studies using wearable devices to assess HRV have correlated decreased cognitive function, bouts of illness, and increased physiological stress associated with a lower HRV (35). However, this has had limited application in hEDS patients due to limitations in the exact mechanism and prevalence of AD within this patient population (10). Assessing overall HRV in patients with chronic conditions is critical for symptom management and overall quality of life, as lower HRV and associated AD have been associated with poorer mental health outcomes, increased mortality, and disease morbidity over time (16, 36).

HRV is described via multiple metrics, including time domains, frequency domains (or signal energy in frequency bands), and non-linear metrics (17). Frequency domains are a span of frequencies where the contributions to the behavior of these cyclical events, such as heartbeats, often referred to as a signal, can be defined as a function of frequency. Frequency, measured in hertz (Hz), is the number of occurrences of cyclical events per unit of time. One method to assess HRV via both time and frequency domains simultaneously is using wavelet transformation-based analysis. By using wavelet transformation methods, representations of changes in HRV can be utilized to correlate changes in both frequency and time domain features to periods of increased physiological stress, manifesting as increased autonomic dysfunction. Therefore, this pilot study aims to understand the intra- and interindividual HRV utilizing wavelet transformation methods in autonomic function associated with high and low-GI-symptom days in individuals with hEDS using wearable device beat-to-beat heart rate data to assess if HRV can be predictive of GI symptom fluctuations.

## 2 Materials and methods

### 2.1 Study participants

Thirty participants were recruited for this study through direct recruitment from a previous hEDS study (37) or outreach via social media and email listservs. Participants were eligible if they were. Aged 18 or older, of any sex or race, met the 2017 diagnostic criteria for an hEDS diagnosis, were willing to comply with all study measures, and were willing and able to use wearable device technology, including an associated smartphone application. Participants were excluded if they had the following conditions known to alter autonomic function: (1) a current medical diagnosis of specific conditions with significant effects on autonomic function such as POTS; diagnosed by tilt table test or cardiological evaluation, untreated sleep apnea, pregnancy, or any primary dysautonomia diagnosis such as neurocardiogenic syncope, familial dysautonomia, multiple system atrophy, or pure autonomic failure; (2) had a cardiac implant used for cardiac rhythm maintenance; (3) were pregnant or intending to become pregnant over the study period, and (4) had any known allergic reactions to using wearable devices. Excluding patients with POTS and other known diagnosed autonomic conditions reduced eligible participation within this pilot but tested the feasibility of capturing relationships between HRV that would be masked by autonomic failure. Eligible participants were asked to consent to all parts of the study, including self-reporting survey data, monthly check-ins, a quarterly orthostatic test, and 24-h continuous heart rate data from the WHOOP wearable device (38). This study was approved for human subject research via the University of Arizona Institutional Review Board as protocol number STUDY00000191.

### 2.2 Study protocol

This was a pilot study assessing autonomic function through the use of a wearable biometric device (WHOOP, Boston, MA, United States) wearable fitness tracker strap (38) or Fitbit (Fitbit is made by Fitbit Inc San Francisco, CA) for 12 months. Four patients who used Fitbit were excluded from further analyses due to differences in collected data. There was no predetermined sample size calculation as this was a pilot study, and the number of participants is in keeping with similar studies of wearable devices and chronic conditions (39).

We conducted an interview to gather information on baseline symptoms, medications, and therapies. Participants were contacted monthly by the study team to report any major changes in self-management that could be used to determine anomalous data for an individual. For the first 6 months of the study, participants completed a daily, weekly, and monthly set of questionnaires measuring various symptoms. This study only used the daily survey for analysis and is described in detail in the following section.

#### 2.2.1 Questionnaire

Participants completed daily questionnaires for 6 months that contained questions about sleep hygiene (time asleep, time awake, number of times awakened during the night, and duration of wakefulness). They also completed 11-point Likert scales asking about symptom severity from None (0) to Extreme (10) for the max value in the preceding 24 h for pain, fatigue, difficulty sleeping, brain fog, anxiety, depression, and GI symptoms. We also asked about any illness or menstruation present. We analyzed only the GI symptom scale in this study for comparison with HRV data.

#### 2.2.2 Biometric data

Biometric data were collected from each participant wearing a WHOOP (Boston, MA, United States) wearable fitness tracker strap (38) for 1 year. Only participants using the WHOOP devices were included in this analysis. Data available include heart rate (HR) (defined as the number of heartbeats per minute) collected once per second, the sleep metrics [e.g., sleep latency, disruptions, rapid eye movement (REM) sleep, and light sleep], and activity metrics, including accelerometer data. This analysis only used the HR data.

### 2.3 Transformations

#### 2.3.1 Gastrointestinal symptom severity

The daily GI severity scales were standardized to *Z*-scores and used to calculate a mean GI symptom severity score. Any days with a GI severity *Z*-score that was 1.5 standard deviations (SDs) away from the participant mean were flagged as high- or “low-symptom days based on the direction of the difference. Biometric data from the low- and high-symptom days were analyzed individually and as pooled data.

#### 2.3.2 Biometric data transformation

The RR intervals collected through WHOOP are not reported at consistent periods across all participants or days. Therefore, we manually calculated RR intervals from device-reported HR data on low- and high-GI-symptom days using the following formula (30):


RR=60s/HR

#### 2.3.3 Heart rate variability

Autonomic function is evaluated using analysis of heart rate variability (HRV). Adaptations seen as fluctuations in heart rate (HR) are essentially the differences in the time between beats (RR interval). Having highly variable responses is consistent with having a normal and functional ANS. The fluctuations in HRV can be mathematically assessed to estimate the overall functionality of the ANS over time in an individual and represent the HR fluctuations around the mean over time. The ANS consists of a system of excitatory and inhibitory signals that can interact synergistically or antagonistically. Thus, the RR signal can be analyzed as a composite of various co-occurring signals that can be mathematically separated or decomposed into distinct frequency domains representing the various ANS functions defined by the number of full signal cycles seen within a discrete unit of time or hertz (Hz).

Previously, the gold standard for analyses of HRV used fast Fourier transform (FFT) based methods (30, 35). However, FFT methods do not allow for the simultaneous analyses of a signal in both the time and frequency domain, precluding the correlation of changes in specific frequency subdomains over time (40, 41). Previous techniques allowed us to look at the distinct frequency signals present within an observed composite signal, but these could not be correlated with the timing of symptoms. Therefore, the current approach is to assess HRV via time and frequency domains simultaneously using wavelet transformation-based analysis. Wavelet transformation methods can create representations of changes in HRV over time and correlate changes in frequency to time, such as identified periods of increased physiological stress or increased symptom severity.

Four recognized HRV frequencies are operating within different bands: ultralow frequency (ULF ≤0.003Hz ), very low frequency ( 0.003< VLF ≤0.04Hz ), low frequency ( 0.04< LF ≤0.15Hz ), and high frequency ( 0.15< HF ≤0.3+Hz ). In general, LF and HF are thought to correspond to activities of the sympathetic and parasympathetic nervous systems, respectively, the VLF corresponds to thermoregulation and vasomotor action, and the ULF is not well understood and is often excluded clinically due to its long duration but is thought to be associated with metabolic or slower endocrine changes (42). Table 1 describes the frequency domains and proposed associated biological mechanisms.

**Table 1:** Heart rate variable frequency domain bands and associated biological processes.

Domain band	Minimum recording intervals (25)	Contributing biological mechanisms	Possible reported outcomes
Ultralow-frequency (ULF) band (≤0.003 Hz)	24 h	Slow-acting biological processes: circadian rhythms, core body temperature, metabolism, and renin-angiotensin system (57)	Psychiatric disorders and sleep dysfunction (58, 59)
Very-low-frequency (VLF) band (0.003–0.04 Hz)	From 5 min to and 24 h	Vasomotor tone involved in thermoregulation and sweating (sympathetic), physical activity, and innervation of the heart (60)	Inflammation, low testosterone, all-cause mortality, and posttraumatic stress disorder (55)
Low-frequency (LF) band (0.04–0.15 Hz)	2 min	Baroreceptor activities [sympathetic control on parasympathetic modulation (55)]	Synchronous fluctuations in blood pressure (61)
High-frequency (HF) band (0.15–0.40 Hz)	1 min	Parasympathetic activity, corresponds with respiratory sinus arrhythmia (55)	Stress, panic, anxiety, and worry (30, 57)

Hz, hertz.

#### 2.3.4 Signal transformations

HRV was decomposed into frequency domains using an 8-level wavelet packet decomposition scheme. This scheme was implemented in the Python 3 Python is an open source license by the Python Software Foundation (43, 44) to produce scalogram plots of signal strength as a function of time for each sampled day of each individual. These plots allow for an assessment of broad trends in activity at HF, LF, VLF, and ULF frequencies across time. In this case, the scale axis of the figures corresponds to 6-h time blocks over 24 h on the time axis to provide a sufficiently large window of data to capture lower frequency domains such as VLF and ULF. These scalograms were inspected for broad trends in the concentration of RR signal strength across time domains by frequency domains. Figure 2 visually represents the data transformations used in this analysis. Power density, or the strength of the RR signal per Hz, was also calculated to validate peaks of scalogram activity across the frequency domain. The transformation methods are detailed in supplemental methods. The ratio of normalized LF/HF power over 24 h. a high LF/HF ratio indicates high sympathetic activity and lower parasympathetic involvement, while a low LF/HF ratio indicates parasympathetic activity (30). Although this remains controversial in HRV analysis due to complexities in balances between the two systems, the impact of testing conditions when analyzing these frequency bands has poor correlation and validation (30, 45).

**Figure 1:**
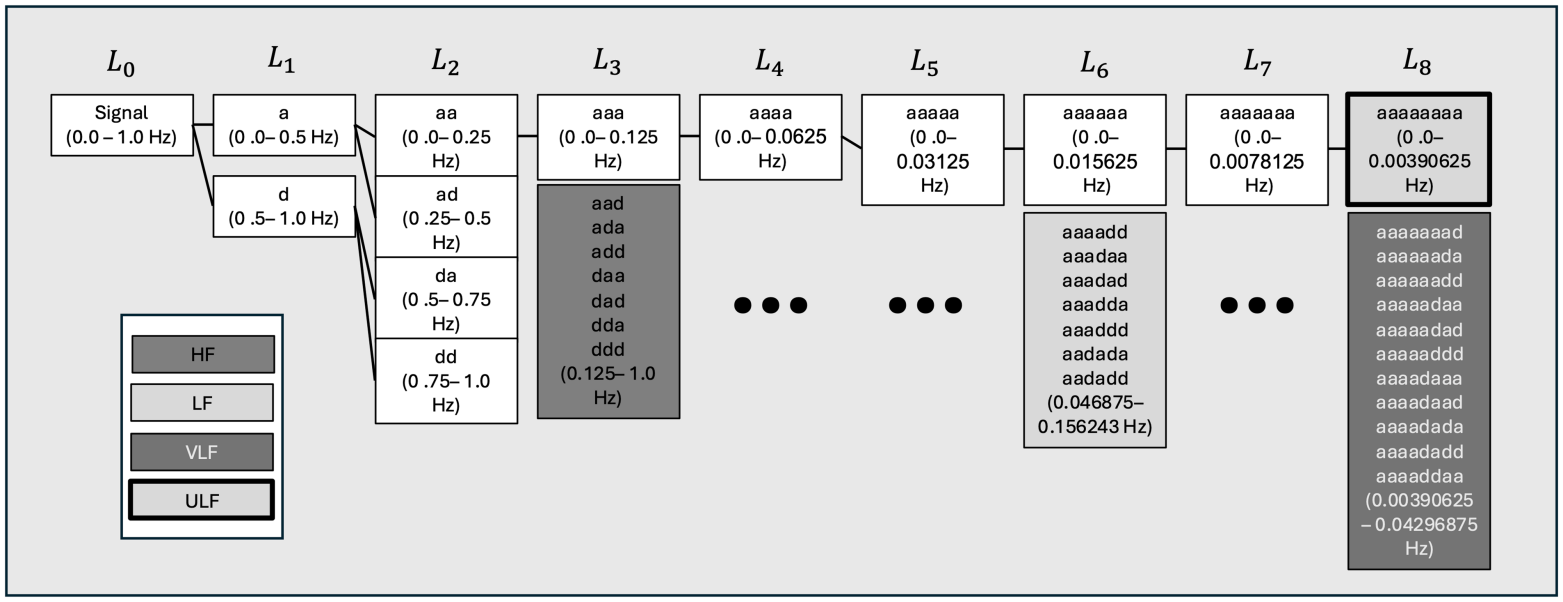
Wavelet packet decomposition tree showing parts of the decomposed heart rate (HR) signal used to reconstruct high (HF), low (LF), very low (VLF), and ultralow (ULF) time domain representations for each individual. A visual summary of the decomposition scheme for each level of wavelet decomposition and shows the process of reconstituting the frequency bands from each heart rate (HR) signal. Hz, hertz; a:d-filtering operations of the wavelet decomposition L-level of decomposition node.

**Figure 2:**
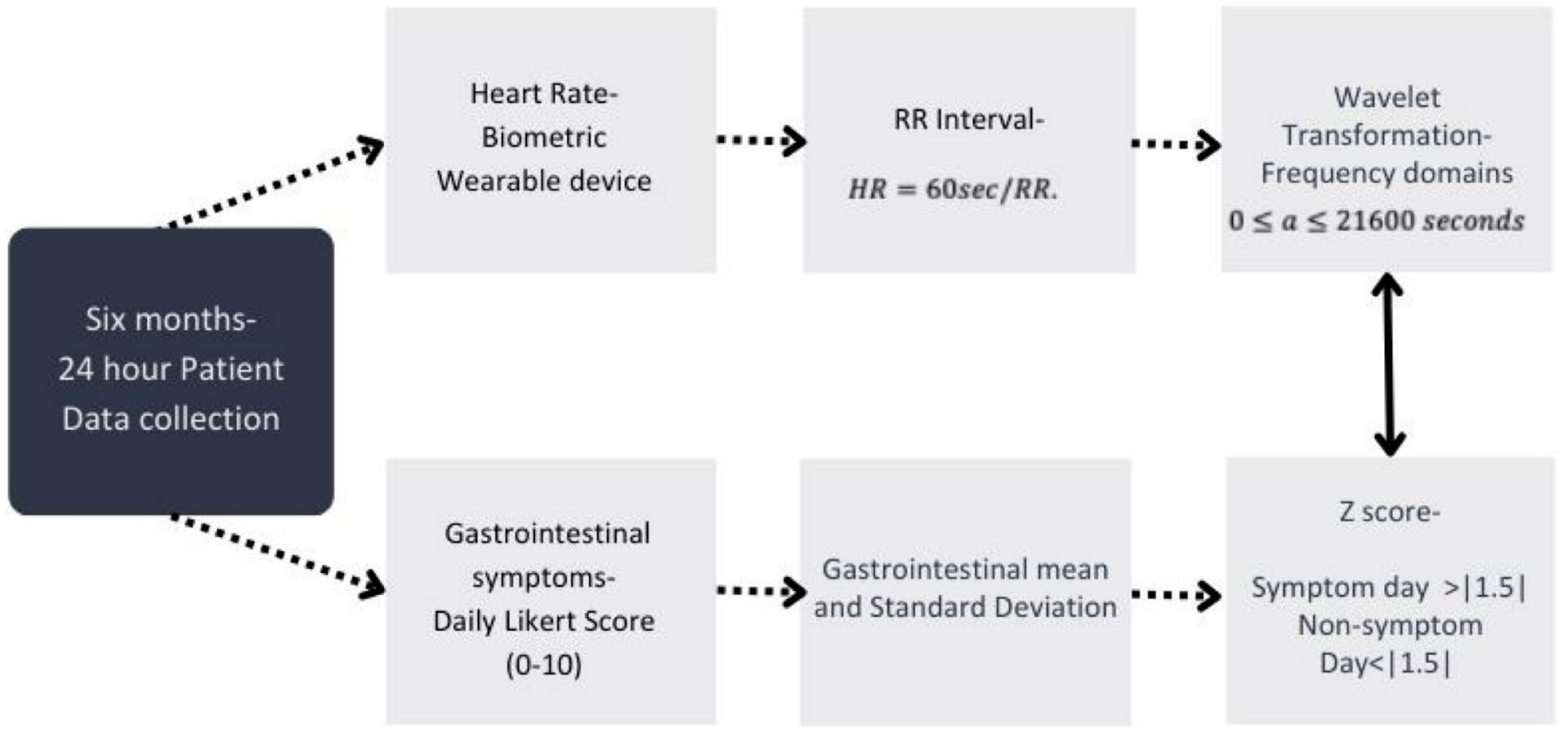
Data processing for all study data used within this analysis and the timeframe of data collection. Timeline of data collection and process of data transformations used for analysis within this study from both the wearable device and self-reported gastrointestinal (GI) survey data collected from participants.

### 2.4 Analysis

#### 2.4.1 Descriptive statistics

Proportions, means, and standard deviations were used to describe the study sample and the number of low- and high-symptom days of data included in the analysis. The frequency domains (HF, LF, VLF, and ULF) that were reconstructed from the wavelet packet decompositions of HRV include RR-mean, RR-variance ( $\sigma^{2})$ , and standard deviations of the RR interval (SDRR) for each frequency domain. The SDRR is a standard HRV metric that captures the RR readings distribution about its mean value. By computing this metric for each time domain by frequency domain generated from the wavelet packet decomposition of the HRV for each sampled date, statistical comparisons of the SDRR about the means for LF, VLF, and ULF subsignals were compared for high- vs. low-symptom days. Consequently, the frequency ranges responsible for the most significant contributions to overall HRV trends could be isolated and identified, This helps to identify periods of symptomatic activity, such as the increased severity of a GI symptom, that assist in identifying upcoming periods of increased AD by tracking changes in the SDRR (30).

#### 2.4.2 Wavelet transformations

**Figure 3:**
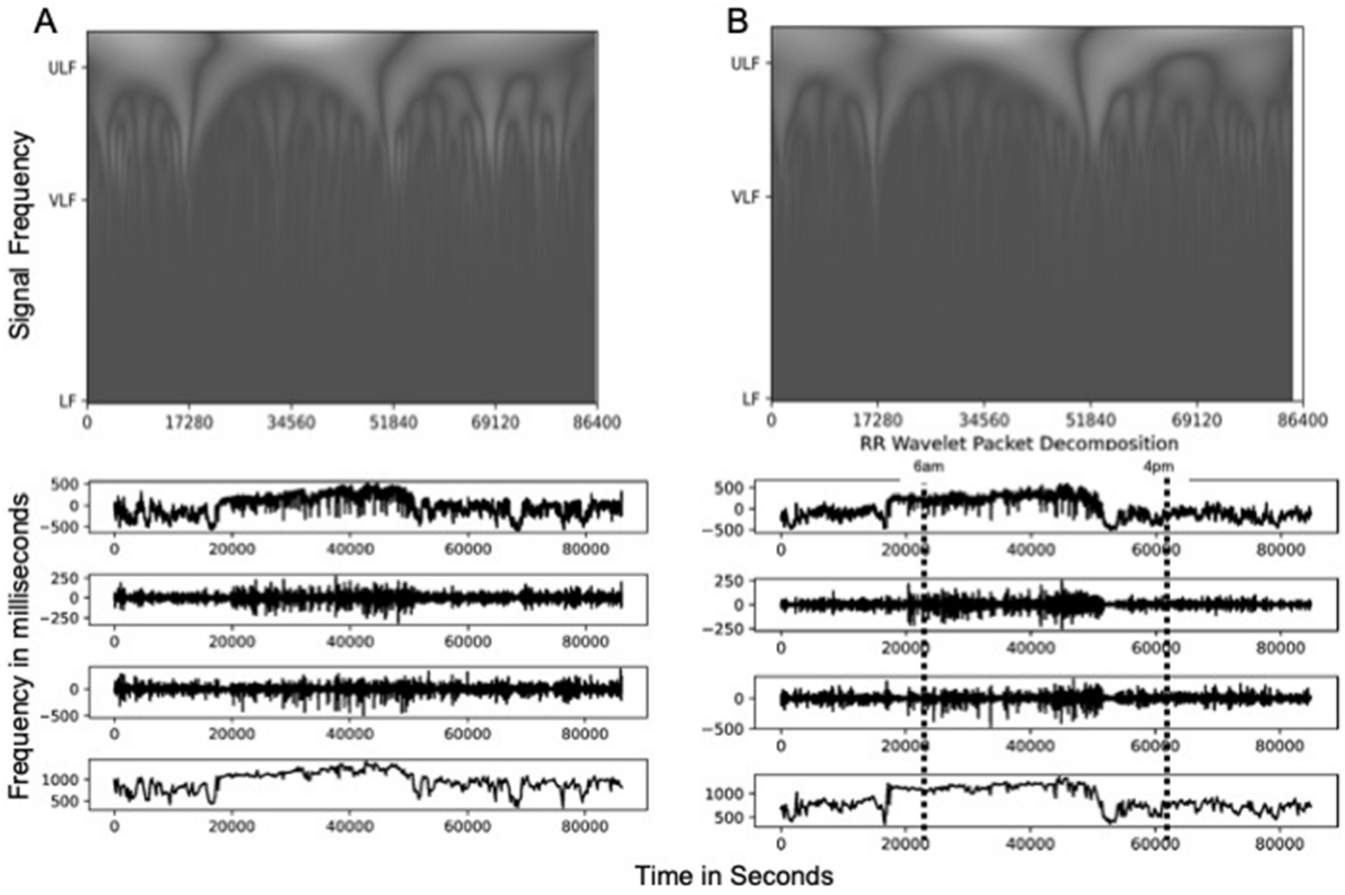
Scalogram of time series plots for low (LF), very low (VLF), and ultralow (ULF) frequency signals and RR Intervals for **(A)** representative participant data and **(B)** wavelet transformed data. These time series representations show periods of increased respiratory rate (RR) levels at roughly 20,000 s from midnight to about 60,000 s, corresponding to conventional times of increased activity of about 6:00 a.m. to 4:00 p.m., with a decrease at about 4:00 p.m. RR levels of greater than 1,000 ms across samples during this period in both the original signal and the ULF component, with peak variations in LF and VLF components at |250| ms and |500| ms, respectively.

A representative example of the scalograms generated with a continuous wavelet transformation of an observed date’s HRV signal is shown in Figure 3. Visualizations for the remaining participant’s dates are available in the supplemental materials. The peaks of these scalograms were compared to the peaks in power density to assess where the most significant signal activity was occurring.

Cross-correlation was performed on R-R and time-domain subsignals for each individual’s high- and low-symptom days to calculate the lag time associated with the maximum cross-correlation value. Here cross-correlation measures the amount of overlap of the plots of two R-R signals. In maximizing the cross-correlation value, you are “sliding” the plot of one signal over the other until the most excellent fit between the two is achieved. The lag is the amount that one signal must be translated to achieve this fit. This is often performed to compare better two signals that may have similar profiles, but which occur at different points in time. This ensures any statistical differences are due to changes in the signal rather than other contributing factors such as effects of changes in daily sleep/wake or inactivity across the population. Aligned RR signals and time-domain subsignals for individuals’ high- and low-symptom days were then paired for statistical comparison.

Differences in observed signal and time-domain subsignal variances, or the square of the SDRR, between sampled days were tested for significance using an *F*-test. *F*-tests were chosen due to the continuous nature of the heart rate data. The test value is calculated by


$$F-\mathrm{value}=\frac{\frac{\sigma_{a}^{2}}{Ndof_{a}}}{\frac{\sigma_{b}^{2}}{Ndof_{b}}}$$

where $\sigma_{a}^{2}$ and $\sigma_{b}^{2}$ are the variances of each variable, and *Ndof*_a_ and *Ndof*_b_ are their respective degrees of freedom or individual readings per tested sample. Here, values from the *F*-test were compared against *p* > 0.01 for hypothesis testing. *F*-values to indicate the ratio of statistically significant sample means (46) were calculated for observed variances of high-symptom days vs. low-symptom days and low-symptom days vs. low-symptom days.

#### 2.4.3 Effects of symptom severity

The results of individual *F*-tests were aggregated, and a population-wide ratio of significant *F*-tests of RR intervals between frequency domains was calculated for the total population and each frequency domain.

The power density calculated the total strength of the HRV signal in the frequency domain. The total average power density of all frequency domains and their SDs were calculated for high- and low-symptom days across the population. Here, power density measures the amplitude of the HRV signal at a given frequency. The average power density for each frequency subdomain can be viewed as a measure of the contribution to the overall amplitude of the HRV measurements of the ULF, VLF, and LF signals. The average power density can be then tested for any statistical differences in the changes in behavior in HR signals, to verify the frequency domain of the majority of the HR signal activity, and to assess for broad trends between high- and low-symptom days.

## 3 Results

A total of 30 participants were recruited, but 4 participants were excluded from the analysis due to the use of a different biometric device that could not be consolidated without additional standardization, resulting in a sample of 26 individuals. These 26 individuals reported 4,615 total daily-GI-symptom days over the 6-month period, the majority of which were within ±1 SD of their overall mean symptom severity score. Approximately 5% of all collected symptom days were classified as either low or high. Of the 230 high- and low-symptom days, 122 days were sampled in the wavelet analysis. Table 2 describes our sample—predominately women (92.3%) of non-Hispanic ethnicity (73.1%). The average age of our cohort was 44.6 years (20–74). Our cohort’s daily GI mean symptom score was 4.73 out of 10, which was normally distributed. High-symptom days (high-GI symptoms) used in this analysis had a mean GI score of 7.59 (±1.28), and low-symptom days had a mean of 2.58 (±1.27). Days with a score of zero, representing no symptoms experienced, occurred on 615 (13.3%) days reported by all participants over the 6-month survey period.

**Table 2:** Demographics, gastrointestinal (GI) symptom severity, and heart rate variability (HRV) characteristics of study participants.

	*N*	%	Mean	SD	*p* < 0.01
Age	26	100	44.6	14.5	<0.001
Gender					<0.01
Female	24	92.3			
Transgender male	2	7.7			
Ethnicity					<0.01
Not Hispanic/Latino	19	73.1			
Hispanic/Latino	3	15.4			
Unreported	5	11.5			
Daily symptom severity	4,615	100			
Quality sleep			3.03	0.856	<0.001
Difficulty sleeping			4.46	2.27	<0.001
Pain severity			5.44	1.60	<0.001
Fatigue severity			5.84	1.64	<0.001
Brain fog/cognition			5.07	1.94	<0.001
Anxiety			3.91	2.52	<0.001
Depression			3.14	2.52	<0.001
Awake occurrences			1.91	1.77	<0.001
Reported days of illness	318	6.8			
GI severity			4.74	1.86	<0.001
Low-symptom days	231	5	2.54	1.27	
High-symptom days	282	6.1	7.59	1.28	
WHOOP sampled days	122	100			<0.001
Low symptom	48	39.3			
High symptom	35	20.4			
Heart sate variability					
RR Interval (ms^2^)	83,959	100	785.99	137.13	
ULF (ms^2^)	83,992	100	785.87	128.90	
VLF (ms^2^)	84,085	100	−0.01	40.18	
LF (ms^2^)	84,085	100	0.00	18.18	

GI, gastrointestinal; ULF, ultralow frequency; VLF, very-low frequency; LF, low frequency; SD, standard deviation.

Table 2 also describes the mean and SD of each daily symptom domain asked in this study. The highest severity of daily symptoms was found in fatigue (5.84 ± 1.64), pain (5.44 ± 1.60), and brain fog (5.1 ± 1.94). The average awake occurrences were reported as 1.91 ± 1.77 times a night for all individuals. Illness was reported in 318 days (6.8%).

Table 3 provides descriptive statistics for the HRV metrics for the low- and high-symptom days. There was no statistical difference in overall HRV metrics comparing the high- and low-symptom days for this population within any frequency domain variance or in the mean between low- and high-symptom days. Only the ULF domain demonstrated a statistical difference in average power density (amplitude) between high- (562109.52 ms^2^) and low-symptom (627435.80 ms^2^) days. The consistency of these time-domain subsignals reconstructed via wavelet transformations, the power density, and the scalogram plots suggest that this study verified wavelet transformations. Power spectrum density analysis showed that amplitudes of HF frequencies occur minimally in this analysis compared to the ULF and VLF due to the more significant time observation period. This made HF contributions to the composite signal minimal and not further analyzed beyond the LF/HF ratio.

**Table 3:** Descriptive statistics, total max correlation, significant *F*-tests, and average power density by frequency domain of total population, low-, and high-symptom days.

	High symptom	Low symptom	Total assessed days
**Descriptive statistics by frequency domain of total population**			
RR mean (ms)	773.49 ±135.05	792.61 ±138.00	785.99 ±137.13
Ultralow frequency mean (ms)	773.39 ±127.08ms	792.47 ±129.57	785.87 ±128.90
RR variance ( $\sigma^{2})$	19648.91	20887.17	20453.37
Low-frequency variance	319.55	367.24	345.39
Very-low-frequency variance	1596.03	1739.79	1683.59
Ultralow-frequency variance	17586.58	18633.12	18274.14
**Total maximum correlation means (ms** ^**2**^ **)**			
High- vs. low-symptom days (ms^2^)	3.94e10		
Low- vs. low-symptom days (ms^2^)	4.15e10		

	**High-/low-symptom day**	**High vs. Low symptom**	**Total**
**Percentage of significant *F*-tests ( *p* < 0.01) of RR interval variance between frequency domains**			
RR Interval	0.55	0.41	0.88
Ultralow-frequency RR component	0.92	0.41	0.89
Very-low-frequency component RR component	0.76	0.53	0.88
Low-frequency component	0.41	0.54	0.88

	**High symptom**	**Power density integral**	**Low symptom**	**Power density integral**
**Average power density means of each frequency domain**				
Ultralow frequency (ms^2^/Hz)	302253.49 ± 204729.12	1047.26244	327609.66 ± 219398.35	1141.11
Very low frequency (ms^2^/Hz)	16023.26 ± 20064.76	515.476423	14499.93 ± 15984.77	470.93
Low frequency (ms^2^/Hz)	275.99 ± 335.02	29.66543	322.27± 393.45	34.72
All frequency domains (ms^2^/Hz)	3773.16 ± 33092.57	1876313.03	3860.71 ± 35374.07	1912562.93

	**High symptom**	**Low symptom**	**All assessed days**	
**LF/HF ratio**				
	5.92 ± 1.28	6.6 ± 1.44	6.30 ± 1.42	

ms, milliseconds, Hz, hertz.

HRV is highly individualized and, therefore, it is not surprising that the calculated variances and means for the total RR and constructed frequency domains did not differ from the overall total between high- and low-symptom days. However, we did find differences in the variance of HRV and frequency domains between high- and low-symptom days at the individual level. *F*-test statistics comparing sampled high- and low-symptom days in individual participants demonstrated statistically significant patterns at *p* < 0.01 with the maximum cross-correlation values (3.94e10 ms^2^ high vs. low and 4.15e10 ms^2^ for low vs. low) occurring at a mean time of 0.0 ( $\bar{\tau }=0.0$ ) (Table 3).

The most significant ratio of successfully aggregated *F*-tests is found in the high-symptom vs. low-symptom sample days for the VLF and ULF domains at ~76 and ~92%, respectively, suggesting a strong correlation between changes in HRV at these frequencies for symptom expression (Table 3). Comparison of variances on low-symptom days tested against high-symptom days yields weakly correlated results, with the majority of the ratios for these tests hovering at approximately 50% across the different frequency bins.

The frequency domain average power density for HRV and ULF, VLF, and LF time domain signals are summarized in Table 3. Higher signal strength (power density) differences were found in the ULF HRV in both the low (327609.66 ± 219398.35 ms^2^/Hz) and high (302253.49 ± 204729.12 ms^2^/Hz) GI-symptom days, as compared with all other frequency domains. Statistically significant differences were only observed in LF (0.08) HRV. The LF/HF ratio was calculated for high- and low-symptom days, and all days were assessed via the frequency domain. High-symptom days reported a ratio of 5.92 ± 1.29; however, low-symptom days and all days assessed reported 6.64 ± 1.45 and 6.30 ± 1.42, respectively.

## 4 Discussion

Symptoms associated with AD have a disproportionate effect on the quality of life for individuals living with chronic illness. These frequently begin with complaints of OI and correlate with perceptions of overall health and symptom exacerbation directly. Specifically, AD is frequently identified in people with hEDS, and the overwhelming majority of people with hEDS report some degree of OI (7, 10, 12–14). Clinically, the standard for assessing autonomic function uses HRV, or the changes in time intervals between consecutive heartbeats, from 24-h Holter monitor ECG data (30), but this is costly and can only be done in short durations. Therefore, we sought to determine the feasibility of using commercial biometric wearable devices to detect subtle changes in autonomic function and to seek to associate that with changes in subjective GI symptom severity. Our study demonstrated that determining HRV patterns using wearable devices in a population with chronic illness is feasible despite their inherent limitations compared with their clinical gold-standard counterparts.

Celletti et al. found that baroreceptors in people with hEDS were more sensitive to changes in pressure, about 30% of individuals could not coordinate their breathing sufficiently to complete the Valsalva maneuver, and none of the participants could complete the sustained handgrip activity (13). Baroreceptor activities are associated with the LF band, and our data showed a significant average difference in power density means between low- and high-GI-symptom days in the LF band.

Cross-correlation between samples is intended to remove contributions of external causes of variability. The minimal lag time between signals during cross-correlation suggests the observed effects are associated with physiological changes. The population level means of the maximum cross-correlation values for each individual were slightly different ( ±~5% ) showing differences in absolute signal behavior values are likely localized within the sampled signals or found in different sample features like variance and standard deviation.

The scalogram and power density calculations demonstrate the critical role of the ULF in shaping the overall HRV trends. The ULF band is less understood than the other bands of HRV primarily due to the previous transformation methods and shorter duration of HRV measurements, ~5–10 min, corresponding to the rapid changes initiated by the nervous system in the LF, VLF, and HF bands. The ULF signal cycle fluctuation could occur in an epoch as long as 24 h and, therefore, is thought to be associated with circadian and neuroendocrine responses in homeostasis. Our data demonstrate that the high variability in ULF and VLF are associated with high-symptom days for 92 and 76% of all F-Tests but show no difference when differentiating low-symptom days from high-symptom days. These domains are associated with slower, longer-term biological responses like thermoregulation, metabolism homeostasis, circadian rhythms, and inflammatory processes, all of which are frequently reported as dysfunctional in people with hEDS (47). This suggests that the GI symptoms are spiking (>1.5 standard deviations) in symptom severity, resulting in a ULF/VLF response.

Weaker and less predictive relationships were found when assessing other frequency domains, particularly the VLF and the LF, suggesting that signals derived from VLF and LF data do not strongly correlate with the expression of symptoms within this study. More specifically, the means of zero for both the VLF and LF bands suggest they only have minimal contributing *hz* power to the total frequency domain for HRV over the 24-h period. This result could be from the need for higher sampling rates to track these frequency domains, or they could correlate better with other symptom domains not analyzed within this study. Future studies will focus on increasing the sample size to assess these frequencies and include additional symptom domains. The LF/HF ratio also did not show the expected results as a higher ratio was found on low-symptom days compared to high-symptom days, although this study found a higher ratio range in all days compared to studies of healthy individuals where a range is expected to fall within 1–2 (48). However, the LF/HF remains controversial and has had poor predictive value in other biometric device studies (49) despite being frequently reported in many HRV clinical analyses.

hEDS patients experience intermittent and frequent symptoms associated with AD that make everyday-symptom management difficult and contribute to poor overall quality of life (16). Moreover, the lived experience of people with hEDS and the overall impact of the condition have been associated with increased anxiety and depression, poor quality of life, and increased functional disability (50). Few self-management techniques have been validated for hEDS patients (45), and lack of clinical knowledge of hEDS and barriers in accessing the necessary clinical specialists have made effective symptom management challenging (51). OI in this population is well documented (10, 13), but no existing clinical or static tests predicted its occurrence (13). Disease burden for AD, GI, and orthostatic complaints is correlated with reduced perception of global health and increased reports of pain (12). Our cohort within this study demonstrated a high average daily burden of many of these other symptoms associated with AD, including brain fog, pain, and fatigue. These symptoms can often be interrelated and cumulative but are often overlooked or undermanaged in clinical care (51). Therefore, identifying periods of increased symptom severity to mitigate bouts of increased functional disability is critical for this population.

Self-management strategies for GI symptoms in hEDS patients are currently underdeveloped. The majority of clinical management relies profoundly on trial-and-error methods of symptom management, predominately based on dietary changes (8, 9, 62). The limited self-management techniques and decreased quality of life associated with GI symptom severity may increase the naturally occurring reduction in HRV as a patient ages (52) and thereby increase the risk of additional comorbidities associated with AD. Additionally, GI symptoms are linked to increased anxiety, which directly relates to the severity of their symptoms and may prohibit patients from seeking alternative management techniques, increasing the severity of their symptoms and increasing their overall long-term AD (53). This study further suggests utilizing biometric devices and HRV analysis may provide a clinical alternative to nutrition-based patient-guided GI dysfunction management.

Some limitations of this study do exist. Symptom domains were based on the self-reported GI data from each participant. This self-reported Likert-scale data can depend on recall bias among our cohort, although it is collected daily, long-term recall is not required. Variability of the GI symptoms within the 24-h periods was not assessed and may impact HRV on a shorter time scale. Additionally, this study only presents dysfunction in one symptom domain, and results may change when multiple morbidities are considered in relationship with HRV. Continuous ECG data provides more precise sampling rates than wearable devices, yielding a higher resolution of raw data that could then be converted to instantaneous reading (54); however, clinical studies rarely consider the ULF band. The biometric devices such as the WHOOP strap (38) used in this study are limited in the frequency of measurements but demonstrate the ability to collect sufficient reliable readings for longer term HRV metrics, especially in longitudinal studies precluding the use of ECG. Our wavelet analysis is still limited by a specific method of HR data generation and the likelihood of user-introduced error (55, 56). Suboptimal band placement, neglecting to charge the device, and false data from environmental or behaviorally induced noise, such as increased respiratory rates during exercise, could all potentially increase error. Finally, the small sample size and lack of inclusion of participants with POTS, while appropriate for a pilot study, will require study replication in a larger and more clinically diverse cohort.

Despite these limitations, the accessibility and relatively low cost of biometric devices such as the WHOOP strap allow for increased feasibility in research for developing self-management assessments for hEDS patients. AD in many hEDS patients represents a significant increase in functional disability, and research into models with the ability to predict symptom activity using biometrics may assist with patient self-management and increased quality of life.

## 5 Conclusion

This study shows a relationship between GI symptom exacerbation and HRV in hEDS patients, specifically in the very and ultralow frequency bands. Changes in HRV are associated with chronic stress, but few studies have attempted to define associations between symptomatic vs. asymptomatic periods caused by functional disease and changes in HRV. Identifying variations within these time domains should assist in creating predictive models that allow for increased self-management by hEDS patients. To establish these predictive models, future studies should also include additional symptom domains that may impact the relationships between HRV and AD, including sleep disorders, fatigue, and pain. Further analyses of longer AD periods compared to low to non-symptom activity days within a wavelet frequency domain analysis will also be explored in the future studies. Finally, this study excluded participants with POTS and other individuals with known altered autonomic dysfunction to determine if this approach was viable in individuals with intermittent AD. Future studies should apply the methods we have developed to larger participant groups and could compare results in people with and without a POTS diagnosis.

## Data Availability

The raw data supporting the conclusions of this article will be made available by the authors, without undue reservation.
