# Supplementary material for: Tracking changes in autonomic function by coupled analysis of wavelet-based dispersion of heart rate variability and gastrointestinal symptom severity in individuals with hypermobile Ehlers–Danlos syndrome
Source: Front Neurol. 2025 Jan 15;15:1499582. doi: 10.3389/fneur.2024.1499582 (PMC11778341; doi:10.3389/fneur.2024.1499582)
Supplement: Supplementary file 1 [file Data_Sheet_1.docx]

# Technical Supplement

# Introduction

# Biometric data transformations

### Heart Rate (HR)

WHOOP straps have a sampling rate of 100 readings per second (100 Hz) and HR datapoints were down sampled and output every minute (1 Hz), yielding 86400 data points per sampled day. This output rate corresponds to a Nyquist frequency of 0.5 Hz, putting the cutoff point for meaningful analysis for this study above the lower bounds of what is considered to be the highest frequency bin of HR data (0.15 Hz +).

### Transformations

Evaluation of autonomic function via analysis of heart rate variability (HRV) uses the multiple measurements including the R-R interval. The autonomic system (ANS) is continually adapting to internal and external stimuli to maintain homeostasis. Adaptations are seen as fluctuations in heart rate (HR) and having highly variable responses is consistent with having a normal and functional ANS. These fluctuations in HRV can be mathematically assessed to estimate the overall functionality of the ANS over time in an individual.

Calculating the time interval between beats (R-R interval ) intervals for every sampled time creates a signal (i.e. the R-R signal) that is the basis for deriving measurements of both time and frequency of HRV(25,34). The R-R signal is subdivided into four frequency subdomain groups: ultra-low frequency (ULF $\leq0.003 Hz$), very low frequency ($0.003<$ VLF $\leq0.04 Hz$), low frequency ($0.04<$ LF $\leq0.15 Hz$), and high frequency ($0.15<$HF $\leq0.3+ Hz$). Although research into the exact relationships between the frequency subdomains and biological processes are theoretical (30), **Table 1** describes the subdomain and proposed associated biological mechanism. Previous analyses of HRV using frequency domains relied on Fast Fourier Transform (FFT) methods which found correlations between chronic conditions and variations in ULF bands (17,20). FFT methods do not allow for the simultaneous analyses of a signal in both the time and frequency domain therefore correlating the behavior of specific frequency subdomains in time represents a shortfall of FFT methods (26,27)

To overcome problems with the FFT methods, we used continuous wavelet transformation to map an HR signal in the time domain onto its corresponding representation across a two-dimensional time plus inverse frequency-domain. Wavelet transformations have been utilized since the 1990’s to assess HRV via electrocardiogram (ECG) but have only had limited applications in wearable devices or research into AD. Continuous wavelet transforms using 2^nd^ order Gaussian wavelet functions in Python (35,36) were used to produce scalograms that plot signal strength as a function of time and inverse frequency, or scale *a,* for each sampled day of each individual. These plots were used to assess broad trends in activity at HF, LF, VLF, and ULF frequencies across time. The range selected for the scale parameter *a* was $0\leq a\leq21600 seconds$. With a sample rate of 1 Hz (3600 seconds/hour), this corresponds to an approximately 6-hour epochs in order to provide a sufficiently large window of data to capture low frequency signal trends such as VLF and ULF. These scalograms were inspected for broad trends in the concentration of R-R signal strength across time and frequency. **Figure 3** provides a visual representation of the data transformations used in this analysis. Power density, or the strength of the R-R signal per *Hz*, were also calculated to validate peaks of scalogram activity across the frequency domain.

R-R signals were also decomposed using an 8-level wavelet packet decomposition scheme. This scheme was implemented in Python 3 using the PyWavelets package using first order Daubechies wavelet functions of two vanishing points (“*db2*”) (35). Approximate and detailed coefficient sets were selected to reconstruct time domain sub signals that corresponded to the LF, VLF, and ULF frequency sub domains to isolate time domain metrics that could be associated directly with physiological behavior at each frequency range based on standard methods (37). **Figure 1** shows the HRV frequency sub domains reconstituted into their corresponding time domain signals.

An analysis of time domain metrics of specific frequency ranges was conducted as sub-signals from each frequency domain (LF, VLF and ULF sub-signals). The standard deviation of the R-R intervals (SDRR) is a standard HRV metric which captures the distribution of R-R readings about its mean value. By computing this metric for each time domain sub-signal generated from the wavelet packet decomposition of the R-R signal for each sampled date, statistical comparisons of the SDRR about the means for LF, VLF, and ULF sub-signals were able to be assessed for symptomatic vs. non symptomatic days. Consequently, the frequency ranges responsible for the greatest contributions to overall HRV trends could be isolated and identified, This helps to identify periods of symptomatic activity, such as the increased severity of a GI symptom, that assist in identifying upcoming periods of increased AD by tracking changes in the SDRR.(25).

## Analysis

### Descriptive statistics

Descriptive statistics were generated from HF, LF, VLF, and ULF time domain sub signals that were reconstructed from the wavelet packet wavelet packet decompositions of the R-R signal. These include RR-mean, RR-variance ($\sigma^{2})$, and standard deviations SDRR for each frequency.

### Individual Differences

A representative example of the scalograms generated with a continuous wavelet transformation of an observed date’s R-R signal is shown in (**Figure 2**). Visualizations for the remaining participant’s dates are available in the supplemental materials. The peaks of these scalograms were compared to the peaks in power density to assess where the greatest signal activity is occurring.

Cross correlation was performed on R-R signals and time domain sub signals for each individual on symptomatic vs. non symptomatic days to calculate the lag time associated with the maximum cross correlation value. This ensures any statistical differences are due to changes in the signal rather than other contributing factors such as effects of changes in daily schedule (i.e. differences in sleep/wake time, periods of activity) across the population. Aligned R-R signals and time domain sub-signals for individual’s non-symptomatic and symptomatic days were then paired for statistical comparison.

Differences in observed signal and time domain sub-signal variances, or the square of the SDRR, between sampled days were tested for significance using an F-test where the test value is calculated by

$$F Value= \frac{\frac{\sigma_{a}^{2}}{{Ndof}_{a}}}{\frac{\sigma_{b}^{2}}{{Ndof}_{b}}}$$

Where $\sigma_{a}^{2}$ and $\sigma_{b}^{2}$ are the variances of each variable, and Ndof_a_ and Ndof_b_ are their respective number of degrees of freedom or number of individual readings per tested sample. Here, values from the F test were compared against p > 0.01 for hypothesis testing. F Values to indicate the ratio of statistically significant sample means (38) were calculated for observed variances of high symptomatic dates against low symptomatic dates.

### Group Differences

The results of individual F tests were aggregated, and a population wide ratio of Significant F-tests of R-R Intervals between frequency domains were calculated for the total population and for each frequency domain.

The total strength of the R-R signal in the frequency domain was calculated by the power density. The total average power density of all frequency domains and their standards deviations were calculated for symptomatic and non-symptomatic days across the population. Here, power density is as a measure of the amplitude of the R-R signal at a given frequency. The average power density for each frequency sub domain can be viewed as a measure of the contribution to overall amplitude of the RR measurements of the ULF, VLF, and LF signals. This tests for any statistical differences in the changes in behavior in HR signals and to verify the frequency domain most signal activity is occurring in, and to assess for broad trends between high symptomatic and low symptomatic days.

1. **Results**

**Table 3** shows the mean, variance, standard deviation and average power for each frequency on high symptomatic and low symptomatic days. Mean values for R-R intervals remained steady across high symptomatic and low symptomatic (773.49 ± $135.05$ vs. 792.61 ±$138.0$) sampled days for both the study participant and the population level. This pattern was seen across the signals for each of the constructed time domain sub signals at the LF, VLF, and ULF sub frequencies. The average HRV for ULF was 785.87 ± 18274.14. The LF and VLF mean values were 0.0 ± 345.39 and -0.01 ±1683.59 respectively. This is suggests that LF and VLF sub frequency contributions to the overall behavior trends of the R-R signal across the date are negligible and do not contribute to the overall power of the total R-R signal in this study. This is consistent with scalogram and power density calculations, where the highest peaks of average power density across the population was observed in the ULF frequency sub domains in both the high symptomatic (562109.52ms^2^) and low symptomatic days (627435.80 ms^2^). The consistency of these time-domain sub signals reconstructed via wavelet transformations, the power density and the scalogram plots suggest that wavelet transformations in this study were verified.

The calculated variances for the total RR (19648.91, 20887.17, 20453.37) and the constructed LF (319.55, 367.24 345.39), VLF(1596.03, 1739.79, 1683.59) , and ULF (17586.58, 18633.12, 18274.14) show similar values at the symptomatic, non-symptomatic and population level, respectively. The differences in the variance of RR and its constituent sub signals calculated from symptomatic to non-symptomatic days was much greater for each individual. At the individual level, when the variances for shigh ymptomatic dates were tested for significance using the F test statistic against sampled low symptomatic days, statistically significant patterns of difference emerged. Across the population, the maximum cross correlation values (3.94e10 ms^2^) high symptomatic vs. low symptomatic and 4.15e10 ms^2^ from low symptomatic vs. low symptomatic) were found to occur at a mean time of 0.0 ($\bar{\tau}=0.0$). This suggests differences in the cross-correlation value were not likely due to effects related to differences in schedule or participant behavior. The population level means of the maximum cross correlation values for each individual’s high symptomatic vs. low symptomatic and low symptomatic vs. low symptomatic days were shown to be only slightly different ($\sim5\%$). This suggests that overall, differences in absolute values of signal behavior were likely localized within the sampled signals or found in different sample features like variance and standard deviation.

The greatest ratio of successful tests is found in the high symptomatic vs. low symptomatic sample days for the VLF and ULF domains at ~76% and ~92%, respectively, suggesting a strong correlation between changes in R-R components at these frequencies for symptomatic expression. Low symptomatic variances tested against high symptomatic yield more weakly correlated results, with most ratios for these tests hovering approximately 50% across the different frequency bins.

The frequency domain features of average “power” density for the observed RR signals and their associated ULF, VLF, and LF time domain sub-signal are summarized in **Table 3**. For each frequency range the average power density measured in ms^2^/Hz, their associated standard deviation, and their total power calculated as a definite integral of the power density across each frequency subdomain are shown. Higher density differences were found in the ULF in both the low symptomatic (327609.66 ±219398.35 ms^2^/Hz) and high symptomatic days (302253.49 ±204729.12 ms^2^/Hz) No statistically significant difference was observed in these values between high symptomatic and low symptomatic days except for in the LF (.08).
